# Successful en bloc venous resection with reconstruction and subsequent radiotherapy for 2 consecutive recurrences of intravenous leiomyoma—a case report

**DOI:** 10.1186/s12885-015-2045-8

**Published:** 2016-01-06

**Authors:** Ying Zhang, Leslie H. Clark, Xiugui Sheng, Chunxiao Zhou

**Affiliations:** Department of Gynecologic Oncology, Shandong Cancer Hospital and Institute, 440 Jiyan Road, Jinan, 250017 China; School of Medicine and Life Sciences, Shandong Academy of Medical Sciences, University of Jinan, Jinan, China; Division of Gynecologic Oncology, University of North Carolina at Chapel Hill, Chapel Hill, NC USA; Lineberger Comprehensive Cancer Center, University of North Carolina at Chapel Hill, Chapel Hill, NC 27599 USA

**Keywords:** Intravenous leiomyomas, Iliac vessel reconstruction, Radiotherapy

## Abstract

**Background:**

Intravenous leiomyomas are a rare variant of uterine leiomyoma. Although histologically benign, these tumors are associated with a poor prognosis due to propensity for metastasis, high recurrence rate, difficulty of obtaining complete resection, and frequent extension into and along major veins.

**Case presentation:**

We describe a 43-year-old patient initially presenting with lower abdominal pain. Clinical examination revealed a large right pelvic mass that was shown by computed tomography (CT) to surround the right external iliac vein, right common iliac vein and distal inferior vena cava. The patient had a history of total abdominal hysterectomy with bilateral ovarian cystectomies for uterine leiomyoma approximately 3 years prior to her presentation. Her past surgical history also included removal of an ovarian endometriosis cyst and right hydrosalpinx. The patient underwent an exploratory laparotomy. Operative findings included complete occlusion of the right iliac vessels and distal vena cava by a large tumor that filled the pelvis and extended to the level of the right kidney. The mass was resected en bloc with the involved veins and synthetic vascular grafts were placed. This highly technical procedure was complicated by hemorrhage requiring a total of 32 units of red blood cells and 2.0 L of plasma. Pathologic examination confirmed intravenous leiomyoma. On Immunohistochemical staining, the tumor cells were positive for CD32, CD34, Vimentin and smooth muscle actin. Eight months after this procedure, the patient again presented with an abdominal mass. She was diagnosed with a pelvic recurrence and noted to have intravascular extension into the left iliac vein and inferior vena cava. For this tumor she underwent radiation treatment with three-dimensional conformal radiation therapy (total dose 4500 cGy). The tumor gradually decreased in size during follow-up and became undetectable by CT.

**Conclusions:**

Surgical excision is the mainstay of treatment of intravenous leiomyoma. Radiation therapy may be an effective alternative in patients with unresectable disease or poor surgical candidates.

**Electronic supplementary material:**

The online version of this article (doi:10.1186/s12885-015-2045-8) contains supplementary material, which is available to authorized users.

## Background

Leiomyomas are the most common type of uterine neoplasm; however, there are several less common variants of leiomyomas. Angioleiomyoma or intravenous leiomyoma (IVL) are a rare variant that originates from mesenchymal tissue. IVL was first described in 1896 by Birch-Hirschfeld. Although it is a benign tumor, IVL has malignant biologic behavior making its management difficult. Because IVL is often diagnosed after it has metastasized to other locations, it generally has a poor prognosis [1]. The tumor can progress to involve the iliac veins, the inferior vena cava (IVC), and even the right atrium [2].

Here we present a rare case of uterine IVL with two recurrences. Our patient was treated with multiple operations, including an extensive resection with grafting of the iliac veins, as well as radiation therapy in order to obtain tumor control. We are the first authors to present the use of radiation therapy for this indication.

## Case presentation

The patient is a 43-year-old woman who presented with a large pelvic mass and was admitted to Shandong Cancer Hospital and Institute in June 2006. Her only complaint was lower abdominal pain. Pertinent past medical and surgical history include: benign hypertension for 2 years, prior cesarean delivery, prior hysterectomy, and inferior vena cava filter placement approximately three years prior for history of venous thrombus.

The patient reported undergoing a total hysterectomy and bilateral ovarian cystectomies for uterine fibroids 3 years prior to this presentation at a local hospital. Pathologic review of this specimen shows uterine IVL based on the Immunohistochemical staining profile and <5 mitotic figures per 10 high-power fields. Additionally, in April 2006, three months prior to her current presentation, the patient was diagnosed with a pelvic mass with complaints of lower abdominal pain and underwent a resection of an ovarian cyst and right salpingectomy. The histopathological assessment of the specimen was an ovarian endometriosis cyst and right hydrosalpinx.

At the time of presentation, gynecologic bimanual exam revealed a palpable mass in the right pelvis, measuring approximately 10 cm × 10 cm × 8 cm. It was firm and non-tender to palpation. The mass appeared to invade the rectovaginal space and extend beyond the pelvis into the abdomen.

**Fig. 1:**
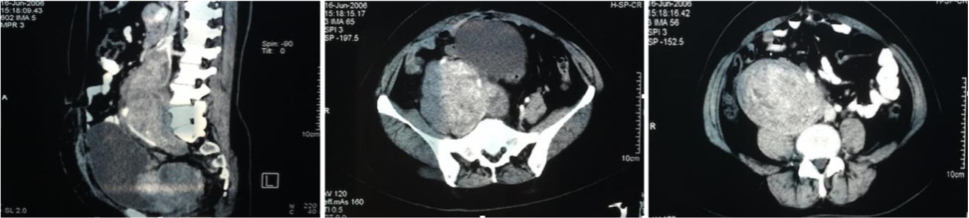
June 16, 2006. A large soft tissue mass with dumb-bell shape and partial cystic degeneration, measuring 25 cm × 13.5 cm × 7 cm. The mass was enlarged since the prior operation 2 months previously. It surrounded the right external and internal iliac veins, right common iliac vein and distal inferior vena cava. It was close in proximity to the right common and internal iliac arteries and aorta up to the level of the right kidney

Contrast-enhanced computed tomography (Fig. 1) of the abdomen and pelvis demonstrated a large soft tissue mass with partial cystic degeneration, measuring 25 cm × 13.5 cm × 7 cm. The mass was noted to be surrounding the right external and internal iliac veins, right common iliac vein and distal IVC. It was in close proximity to the right common iliac artery and abdominal aorta up to the level of the right kidney. Doppler ultrasound showed that tumors blood supply was likely originating from the iliac artery.

Based on the physical exam and radiological findings, combined with the medical history, a preliminary diagnosis of intravascular leiomyoma was made with extension into the right iliac vein and IVC. The recommended treatment was surgical resection. The patient was counseled that surgery would include en bloc resection of the mass with vascular grafting of the distal IVC and common iliac vessels as needed.

Surgery was performed under general anesthesia on July 5, 2006. Upon abdominal entry, two large masses were noted in the right retroperitoneum. One mass was abdominal and measured approximately 20 cm × l8 cm × 10 cm. This mass encased the distal IVC,the right common iliac vein and right external and internal iliac veins. It was closely adherent to the right internal iliac artery. The other mass measured approximately 10 cm × 10 cm × 8 cm and was adherent to the bladder wall, pelvic floor, and right iliac veins. Upon further exploration, the two masses were connected as a single tumor in a dumb-bell shape. The right external and internal iliac veins, right common iliac vein and IVC were completely occluded by the tumor. The right internal iliac and common iliac arteries were dissected off the tumor. The right internal iliac vein was ligated distal to the tumor. The tumor was dissected off the pelvic sidewall. Next the distal IVC and right external iliac vein were ligated. Finally, the left common iliac vein was ligated to free all venous attachments from the tumor and obtain a surgical margin. The tumor had a predominant blood supply from a single pedicle which was divided and the tumor removed en bloc. The en bloc resection included the distal IVC with proximal left common iliac vein, entire right common iliac vein, and proximal portion of the right internal and external iliac veins. An 8 mm expanded polytetrafluoroethylene vascular graft (by Bard Medical) was placed to reconnect the distal IVC to the left common iliac vein with 4-0 Prolene suture to restore venous return to the left lower extremity and pelvis. Given that the patient had developed significant collateral flow with no right extremity edema or venous stasis, the right iliac veins were not reconstructed. Removal of the tumor was challenging with an estimated intraoperative blood loss of 5.0 L. In total the patient was transfused 32 units of packed red blood cells and 2.0 L of plasma. Postoperatively the patient was closely observed for distal pulses, evidence of edema, and lower extremity compartment syndrome.

**Fig. 2:**
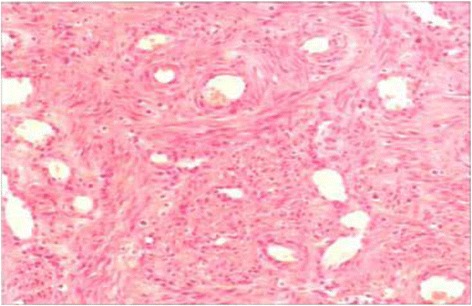
Pathologic diagnosis. The tissue was rich in blood vessels, and tumor cells were arranged around the blood vessels (hematoxylin and eosin, 100×)

Postoperative pathology confirmed this mass to be an intravenous leiomyoma arising from the right internal iliac vein (Fig. 2). Immunohistochemistry shows the endothelial cells to be positive for CD31, CD34, Vimentin and smooth muscle actin. The cells stain negative for cytokeratin with low expression of p53 protein and high expression of progesterone receptor and Bcl-2.

The patient was managed postoperatively on anticoagulation and given nutritional support with intravenous nutrition until able to tolerate oral intake. She ultimately recovered from the operation and was discharged in a stable condition on postoperative day 20.

**Fig. 3:**
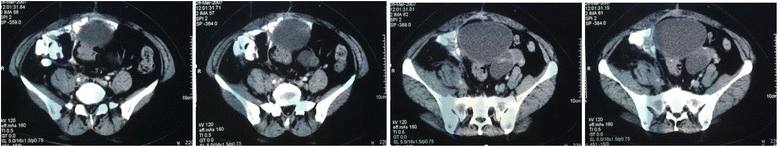
March 26, 2007. There was a soft tissue mass close to the site of the prior tumor near the right internal iliac vessels, measuring 3.5 cm × 4.0 cm × 4.0 cm. Additionally, a left sided mass behind the bladder, measured 3.0 cm × 5.0 cm × 6.5 cm

Eight months following her surgical resection, the patient represented complaining of an enlarging abdomen and frequent urination. She was readmitted to hospital in March 2007. Clinical examination revealed a mass measuring approximately 6 cm × 6 cm in the left pelvis. CT examination revealed a soft tissue mass close to the previously resected tumor involving the right internal iliac vessels, measuring 3.5 cm × 4 cm × 4 cm. A separate mass was also seen posterior to the bladder on the left, measuring 3 cm × 5 cm × 6.5 cm (Fig. 3). This was felt to represent a pelvic recurrence of IVL. The patient was deemed to not be an operative candidate given deconditioning from the prior surgery. Three-dimensional conformal radiation therapy was performed for tumor control. External beam radiation was planned using an ADAC Treatment Planning System, delivered with 15MV X-ray from a Varian 21EX (Palo Alto, CA) and designed from CT scans. External irradiation was 2.0 Gy daily fraction, 4 fractions per week, the total dose of radiation was 4500 cGy.

**Fig. 4:**
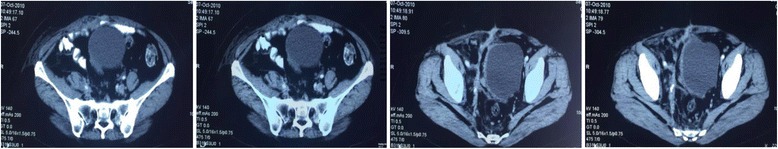
Oct 7, 2010. A pelvic mass to the right of the bladder and rectum measuring 4 .0 cm × 2.0 cm

**Fig. 5:**
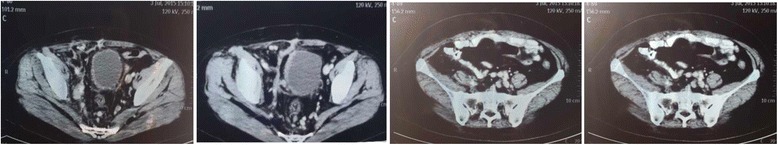
July 3, 2015. No signs of recurrence were detected at follow up

Following radiation therapy, the patient was followed closely. A subsequent CT scan in October 2010 demonstrated a pelvic mass on the right of the bladder and rectum. It was again felt to be surrounding the distal IVC and iliac blood vessels. The largest cross-sectional area was 4.0 cm × 2.0 cm (Fig. 4). This lesion was not further treated, but followed with serial imaging and resolved. At most recent follow-up 8 years after radiation therapy in July 2015 the patient remains without evidence of recurrence on physical examination and CT images (Fig. 5).

## Discussion

IVL is a rare benign neoplasm arising from the vascular smooth muscle [1]. It presents as a painful mass in ~60 % of cases [3]. IVL is the benign hyperplasia of smooth muscle cells, as is seen in uterine fibroids, but it has propensity to grow and metastasize like a malignant tumor. It has been reported to involve the IVC, right atrium, and lungs and may be potentially life-threatening [2]. A patient presenting following a hysterectomy for leiomyoma with a solid abdominopelvic mass on clinical examination or imaging, should prompt providers to consider a recurrence of IVL [4, 5].

In complex cases of IVL, whether a tumor can be completely resected depends primarily on the extent of involvement of associated vessels [6]. Forced detachment can easily damage vessels, especially veins with thin, fragile walls, and result in severe hemorrhage. An incomplete resection can result in the residual tumor cells on the vascular surface recurring in the future. The controversy in these types of cases includes whether to resect and reconstruct the iliac veins, as well as how this should be done. As a result of the development of advanced vascular surgical techniques and vascular grafts, we were able to completely resect the tumor en bloc with the involved vessels, and implant vascular grafts [7, 8].

It was necessary to determine the size and shape of the mass as well as the scope and extent of the involved adjacent organs, especially in relationship to the iliac veins and IVC. This evaluation can be performed using contrast-enhanced CT, magnetic resonance imaging, nuclear magnetic resonance angiography, and digital subtraction angiography [5, 9]. In the presented case, the complex involvement of the mass with critical veins made complete resection of the tumor unlikely, without damaging the associated veins. Therefore, the decision was made to remove the tumor en bloc with the affected veins using vascular grafting. By performing en bloc resection with reconstruction of the iliac vessels, there was an improved chance for optimal cytoreduction, and therefore reduced risk of recurrence with associated improvement in survival [7].

Undertaking such an extensive operation carries significant risks for the patients and surgeons. Careful preparations preoperatively, correct judgment intraoperatively, and close postoperative observation is essential. Success also requires close cooperation among multiple teams ranging from vascular surgeons to general surgeons to anesthesiologists and intensive care unit (ICU) physicians.

The mainstay of treatment for IVL is surgical removal, including cases of recurrence and metastasis. Prognosis of IVL depends on whether the tumor is completely resected [10]. As a result, patients not desiring future fertility should be advised to undergo total hysterectomy. However, up to 30 % of patients can develop recurrence and metastasis after initial tumor resection [10]. Therefore, whether or not the tumor is completely resected, routine postoperative follow-up is necessary for prompt diagnosis and treatment of any recurrences [11]. Some studies suggest that IVL is hormone-dependent making anti-hormonal therapy a possible treatment strategy [12]. Anti-estrogens such as GnRH agonists like Leuprorelin Acetate have been used for treatment of IVL [13]. But the role of anti-estrogens therapy for IVL in general remains unclear [11, 14].

Determining the best curative treatment option for a patient who is not suitable for surgery is challenging. In the present case there were concerns surrounding the use of hormonal therapy in a patient with prior venous thromboembolism. Given that the patient was deemed not to be a surgical candidate due to deconditioning and concerns surrounding use of hormonal therapy, she was counseled by her team and opted for radiation therapy. Ultimately, this strategy was shown to be successful with effective control of the tumor, and its gradual reduction to no visible tumor on her most recent CT (Fig. 5).

It is well-documented that radiation therapy is a commonly used means of treating malignant tumors. There are also case reports of selectively using radiation treatments for benign tumors, especially those with biologic activity mimicking malignancy [15, 16]. While malignant tumors usually display immediate response to radiation therapy, our patient displayed a delayed response to radiation therapy with appreciable decrease in the size of the tumor beginning 3 months after completion of treatment. No residual tumor was seen on imagining 6 months following treatment. We suspect that the overall slow response rate to radiation observed was a result of the relatively slow growth rate of IVL compared to malignancy. Given that radiation relies on DNA damage leading to the inability to replicate, this relatively slow growing tumor took longer to respond to radiation than would be expected for a malignancy. When radiation therapy is applied to benign tumors, it can be used at lower doses. This offers the advantage of fewer side effects, which may be more acceptable to patients, especially in those who are not surgical candidates.

## Conclusion

For IVL, radical surgical excision remains the mainstay of treatment. In cases where dissection of the tumor off involved vessels appears to be challenging or not feasible, consideration should be given to vascular resection en bloc with graft placement in order to obtain a complete tumor resection. In the event of a recurrence or tumor in a patient deemed a poor surgical candidate, radiation therapy is feasible and effective alternative to surgical or medical management for prevention or treatment of recurrence.

## Consent

Written informed consent was obtained from the patient for publication of this case report and any accompanying images (Additional file 1). A copy of the written consent is available for review by the editor of this journal.

## Additional file

Additional file 1:
**CARE Checklist (2013) of information to include when writing a case report.** (DOCX 1527 kb)

## Abbreviations

CT: computed tomography
IVL: intravenous leiomyoma
IVC: inferior vena cava
ICU: intensive care unit

## References

[1] Sahu L, Tempe A, Agrawal A (2012). Angioleiomyoma of uterus. J Obstet Gynaecol.

[2] Zizi-Sermpetzoglou A, Myoteri D, Arkoumani E, Koulia K, Tsavari A, Alamanou E (2015). Angioleiomyoma of the uterus: report of a distinctive benign leiomyoma variant. Eur J Gynaecol Oncol.

[3] Hsu TL, Changchien CC, Huang CC, Lin H (2008). Angioleiomyoma originating from the ovary of an eleven-year-old premenarchal girl. Gynecol Obstet Invest.

[4] Peng HJ, Zhao B, Yao QW, Qi HT, Xu ZD, Liu C (2012). Intravenous leiomyomatosis: CT findings. Abdom Imaging.

[5] Dalainas I (2008). Vascular smooth muscle tumors: review of the literature. Int J Surg.

[6] Anaya-Ayala JE, Cheema ZF, Davies MG, Lumsden AB, Reardon MJ (2011). Concomitant reconstruction of infrarenal aorta and inferior vena cava after en bloc resection of retroperitoneal rhabdomyosarcoma. Vasc Endovascular Surg.

[7] Schneider JR, Sener SF, Barrera E (2008). Combined replacement of infrarenal aorta and inferior vena cava after en bloc resection of retroperitoneal extraosseous osteosarcoma. J Vasc Surg.

[8] Schwarzbach MH, Hormann Y, Hinz U, Leowardi C, Bockler D, Mechtersheimer G (2006). Clinical results of surgery for retroperitoneal sarcoma with major blood vessel involvement. J Vasc Surg.

[9] An JY, Heo JS, Noh JH, Sohn TS, Nam SJ, Choi SH (2007). Primary malignant retroperitoneal tumors: analysis of a single institutional experience. Eur J Surg Oncol.

[10] Lin J, Song X, Liu C (2014). Pelvic intravascular leiomyomatosis associated with benign pulmonary metastasizing leiomyoma: clinicopathologic, clonality, and copy number variance analysis. Intl J Gynecol Pathol.

[11] Fasih N, Prasad Shanbhogue AK, Macdonald DB, Fraser-Hill MA, Papadatos D, Kielar AZ (2008). Leiomyomas beyond the uterus: unusual locations, rare manifestations. Radiographics.

[12] Valdes Devesa V, Conley CR, Stone WM, Collins JM, Magrina JF (2013). Update on intravenous leiomyomatosis: report of five patients and literature review. Eur J Obstet Gynecol Reprod Biol.

[13] Morice P, Chapelier A, Dartevelle P, Castaigne D, Lhomme C (2001). Late intracaval and intracardiac leiomyomatosis following hysterectomy for benign myomas treated by surgery and GnRH agonist. Gynecol Oncol.

[14] Liu B, Liu C, Guan H, Li Y, Song X, Shen K (2009). Intravenous leiomyomatosis with inferior vena cava and heart extension. J Vasc Surg.

[15] Yoneoka Y, Tsumanuma I, Fukuda M, Tamura T, Morii K, Tanaka R (2008). Cranial base chordoma--long term outcome and review of the literature. Acta Neurochir.

[16] Schulz-Ertner D, Nikoghosyan A, Thilmann C, Haberer T, Jakel O, Karger C (2003). Carbon ion radiotherapy for chordomas and low-grade chondrosarcomas of the skull base. Results in 67 patients. Strahlenther Onkol.

